# Commentary: Case Report: a giant liposarcoma of the spermatic cord

**DOI:** 10.3389/fonc.2025.1689457

**Published:** 2025-11-17

**Authors:** Jiro Ichikawa, Tomonori Kawasaki, Masanori Wako, Satoshi Ochiai, Tetsuhiro Hagino, Tetsuo Hagino, Kojiro Onohara

**Affiliations:** 1 Department of Orthopaedic Surgery, Interdisciplinary Graduate School of Medicine, University of Yamanashi, Chuo, Japan; 2 Department of Pathology, Saitama Medical University International Medical Center, Hidaka, Japan; 3 Department of Orthopaedic Surgery, National Hospital Organization (NHO) Kofu National Hospital, Kofu, Japan; 4 Department of Diagnostic Radiology, Interdisciplinary Graduate School of Medicine, University of Yamanashi, Chuo, Japan

**Keywords:** atypical, lipomatous, tumor/well-differentiated, liposarcoma, (ALT/WDLPS), dedifferentiated liposarcoma (DDLPS), myxoid liposarcoma (MLS), MRI

## 1 Introduction

Liposarcomas comprise four subtypes, including well-differentiated, myxoid, pleomorphic, and dedifferentiated (1). They are most commonly located in the extremities and retroperitoneum, with well-differentiated liposarcomas (WDLPSs) being the most prevalent (1). We were very interested in the recent publication by Wang et al., “Case Report: A Giant Liposarcoma of the Spermatic Cord,” and sincerely appreciate the authors reporting this valuable case (2). Our differential diagnoses are i) atypical lipomatous tumor/well-differentiated liposarcoma (ALT/WDLPS) and dedifferentiated liposarcoma (DDLPS) based on pathological findings, and ii) myxoid liposarcoma (MLS) based on accompanying magnetic resonance imaging (MRI). Herein, we present the histopathological and imaging characteristics of ALT/WDLPS, DDLPS, and MLS for further discussion.

## 2 Subsections relevant for the subject

Regarding the MRI findings, T1- and T2-weighted images were unavailable, making it difficult to confirm that all suppressed areas in this case represented adipose tissue. These images should therefore be presented, if possible, for greater diagnostic clarity. Regarding the pathological findings, only low-magnification images were provided. As histological evaluation is crucial for differentiating the four tumor subtypes, high-magnification images should be included to clearly depict the morphological characteristics of the constituent cells.

## 3 Commentary and discussion

### 3.1 DDLPS and ALT/WDLPS

DDLPS and ALT/WDLPS are cytogenetically related, both involving high amplification of chromosome 12q13–q15 (3), which contains genes including MDM2 and CDK4. Dedifferentiation occurs in approximately 10% of ALT/WDLPS cases, with retroperitoneal origin being a risk factor (3). ALT/WDLPS exhibits three histological subtypes, including lipoma-like, inflammatory, and sclerosing (4). The most common, lipoma-like ALT/WDLPS, is characterized by mature adipocytes of varying sizes and hyperchromatic stromal spindle cells in the septa and blood vessel walls. A histological characteristic of DDLPS is the transition from ALT/WDLPS to a predominantly high-grade non-adipocytic sarcoma component (5). This transition is often distinct but can sometimes be gradual, with areas of histological blending (5). The ALT/WDLPS component is minimal in some cases, making identification challenging (5). Dedifferentiated areas exhibit various morphological patterns, often resembling undifferentiated pleomorphic sarcoma or myxofibrosarcoma (5), with stromal composition varying from collagenous to myxocollagenous or myxoid types. Notably, ALT/WDLPS and DDLPS, particularly in the abdominal, retroperitoneal, and spermatic cords, may exhibit extensive myxoid stroma (3). Rarely, dedifferentiated components may be low-grade, resembling fibromatosis, low-grade myxofibrosarcoma, or sclerosing ALT/WDLPS, necessitating careful differentiation (5). Heterologous osseous, cartilage, and myoid differentiation occurs in approximately 5–10% of DDLPS cases (3, 5). We reported a case of DDLPS with leiomyosarcoma-like features showing strong expression of myoid markers such as h-caldesmon, desmin, and α-SMA (6). Expression of MDM2 and CDK4, as assessed by immunohistochemistry (IHC), along with MDM2 amplification detected via fluorescence *in situ* hybridization (FISH), is crucial for distinguishing ALT/WDLPS and DDLPS from other tumors (3). A recent report has shown a correlation between high CDK4 expression and poor prognosis, suggesting that CDK4 may be useful not only for diagnosis but also as a prognostic factor (7). Differentiating ALT/WDLPS from DDLPS is critical because of significant differences in recurrence rates and overall survival (3).

### 3.2 MLS

MLS is the second most common liposarcoma, frequently occurring in the proximal extremities (1). Although similar to ALT/WDLPS and DDLPS, MLS is cytogenetically distinct, characterized by the t(12;16)(q13:p11) translocation producing the FUS-DDIT3 fusion protein (8), with the EWSR1-DDIT3 fusion being a less-common variant (8). Histologically, MLS consists of round-to-oval mesenchymal tumor cells lacking adipocytic differentiation, co-existing with variable uni- or multi-vacuolated lipoblasts (1). The tumor contains a myxoid matrix with chicken-wire capillary networks (8). MLSs with ≥5% round-cell components are classified as high-grade and associated with poor prognosis (1, 9). No specific IHC markers reliably diagnose MLS (8), so differentiation from other round-cell sarcomas requires FISH or polymerase chain reaction. Although FUS and EWSR1 may occur in other tumors, DDIT3 is unique to MLS (8). The 5-year survival rate of MLS is approximately 80%, whereas 20% of cases develop metastases, often extrapulmonary, particularly to bone and the retroperitoneum (a pattern distinct from other sarcomas) (9). Compared to other liposarcomas, MLS exhibits higher sensitivity to chemotherapy and radiotherapy, making this a notable therapeutic feature (9). The key clinicopathological distinguishing features among ALT/WDLPS, DDLPS, and MLS are summarized in **Table 1**.

**Table 1:** Key distinguishing features among ALT/WDLPS, DDLPS, and MLS.

Features	ALT/WDLPS	DDLS	MLS
Age (decade)	4th to 6th	5th to 6th	4th to 5th
Location	Deep	Deep	Deep
Size (mean or median)	>10cm	>10cm	8-12cm
Histology	Lobules of mature adipocyte, fibrous septa; spindle cells showing nuclear enlargement and hyperchromasia within the adipocytic or stromal component, Lipoblast; possible	Transition from ALT/WDLPS to non-lipogenic sarcoma, frequent histological feature of undifferentiated pleomorphic or spindle cell sarcoma, Lipoblast; yes	Abundant, basophilic myxoid stroma and chicken-wire-like capillary networks, lack of atypia and mitotic activity, Lipoblast; rare or absent
Immunohistochemistry	MDM2/ CDK4	MDM2/ CDK4	NA
Molecular features	MDM2/CDK4 amplification	MDM2/CDK4 amplification	FUS/EWSR1::DDIT3 rearrangement

ALT/WDLPS, atypical lipomatous tumor/well differentiated liposarcoma; DDLPS, dedifferentiated liposarcoma; MLS, myxoid liposarcoma.

### 3.3 Imaging feature of DDLPS and MLS

When considering prognosis and treatment, the discussion should focus on DDLPS and MLS. A key MRI characteristic of DDLPS is the presence of fat components resembling WDLPS (10). However, fat presence varies, and 73% of cases show no fat (11). Non-fat components often exhibit intermediate T2-weighted signals. In contrast, MLS is characterized by fat components and myxoid elements (10). In MLS, fat components are absent in over 75% of cases, with 30% possessing 0–50% fat and 63.9% showing no fat content (10). Regarding myxoid components, 17% of cases exceed 75% myxoid content, 30% possess 50–75%, and 53% possess less than 50% (10). A higher proportion of myxoid components is significantly associated with lower histological grades (10). Contrast enhancement is more pronounced in DDLPS than MLS (10). These findings make detailed differentiation using T2 fat suppression alone extremely challenging. Introducing T1, T2, and, if possible, contrast-enhanced T1-weighted images would aid differential diagnosis. Furthermore, given the existence of myxoid DDLPS, accurate subtype identification requires combined evaluation of imaging and pathological findings.

## 4 Conclusions

A range of potential differential diagnoses should be considered based on the imaging and pathological findings of the rare spermatic cord liposarcoma recently reported by Wang et al. Lipogenic tumors range from benign to malignant, encompassing numerous disease entities and histological subtypes. Although not elaborated in detail, atypical spindle cell/pleomorphic lipomatous tumors possess imaging characteristics of DDLPS (12) and MLS (13), requiring careful differentiation. Given this complexity, definitive diagnosis should integrate histopathological findings, IHC expression profiles, and FISH results to ensure accuracy. Distinguishing high-grade sarcomas is essential and merits a meticulous approach, as recurrence rates and prognoses vary greatly.
